# The need for hospital-based neonatal palliative care programs in Saudi Arabia

**DOI:** 10.4103/0256-4947.55161

**Published:** 2009

**Authors:** Saleh Al-Alaiyan, Fahad Al-Hazzani

**Affiliations:** From the Department of Pediatrics, King Faisal Specialist Hospital and Research Center, Saudi Arabia

## Abstract

The terms palliative care, supportive care, and comfort care are used to describe individualized care that can provide a dying person the best quality of life until the end. The term “end-of-life care” is also used in a general sense to refer to all aspects of care of a patient with a potentially fatal condition. While the concept of palliative care is not new, it has only recently been applied to the neonatal population. To the best of our knowledge, none of the neonatal intensive care units (NICUs) in Saudi Arabia have adopted a neonatal program for palliative care. We believe the main reason is lack of knowledge of such programs and the fear of being accused of being heartless and cruel by providing comfort care for dying babies. Comfort care begins with the diagnosis of a life-threatening/terminal condition, and continues throughout the course of illness regardless of the outcome. In this perspective, our aim is to introduce these programs for caregivers in the NICUs in Saudi Arabia. For this purpose, we have reviewed the current recommendations in establishing neonatal palliative care programs and discussed some of the social and religious aspects pertaining to this issue.

Advances in obstetric and neonatal care have led to increased survival in premature infants; however, it has been noted that a substantial rise in the postneonatal death rate had accompanied this decrease in neonatal mortality.^1^ The need for palliative care in the neonatal intensive care unit (NICU) is accentuated by the high neonatal mortality rate when compared with mortality among older children. In the US, it has been calculated that 34% of childhood deaths occur in the neonatal period as per the report of the Institute of Medicine (2003); When Children Die: Improving Palliative and End-of-Life Care for Children and Their Families.^2^ They also reported the five leading causes of infant death. These, in descending order, were: (1) congenital malformations, deformations, and chromosomal abnormalities, which accounted for one-fifth of all infant deaths; (2) short gestation and low birth-weight; (3) sudden infant death syndrome (SIDS); (4) newborns affected by maternal complications of pregnancy; and (5) newborns affected by complications of placenta, cord, and membranes. These leading causes differ from one country to another. In a report that included 193 countries, the major causes of neonatal death globally were: infections (sepsis/pneumonia, tetanus, and diarrhea) (35%); preterm birth (28%); and asphyxia (23%).^3^

In the Asir region, Saudi Arabia, the major causes of neonatal mortality were low birth weight (45%), congenital malformations (30.8%), infection (13.6%), and birth asphyxia (7.7%).^4^ A recent single center report from Saudi Arabia involving 79 871 live births and 526 deaths showed that prematurity and its complications were the initial causes of neonatal and postnatal deaths (42%), followed by lethal malformations (36%), and hypoxic ischemic encephalopathy (5%), while 17% of cases died of different disorders.^5^

Hence, dealing with the death of an infant in the NICU is a relatively common problem. Most neonatal deaths occur during the first week of life, so it is mostly the NICU staff confronted with the palliative care of dying neonates. Clinical experience shows that many aspects of care in palliative situations are not well known to the healthcare providers. Its impact on staff can be large, but its impact on families can be immeasurable with lifelong implications. One goal of comfort/palliative and end-of-life care is to minimize avoidable conflicts related to poor communication, cultural misunderstandings, deficient clinical care, and approaches to decision making that fail to assure families that they and the healthcare team are doing their best for the child. Such failures can haunt family members and clinicians long after a child's death.

Therefore, understanding the right process of palliative care by the NICU personnel has a positive impact on families. When such measures are the focus of care, everyone in the NICU stands to benefit, including the newborn patient, his or her family, and the NICU staff. We would like to outline the main pillars of neonatal palliative care and to look at the potentials and the barriers especially to culture sensitivity and spiritual support, when such programs are established at our hospitals.

## What is palliative care?

In the year 2000, the World Health Organization (WHO)^6^ defined palliative care as an approach that improves the quality of life of patients and their families facing the problem associated with life-threatening illness, through the prevention and relief of suffering by means of early identification and impeccable assessment and treatment of pain and other problems, physical, psychosocial and spiritual. Additionally, the Health Resources and Services Administration (HRSA) has set forth the following working definition: Palliative care is patient- and family-centered care. Palliative care is meant to optimize the quality of life by active anticipation, prevention, and treatment of suffering. It emphasizes use of an interdisciplinary team approach throughout the continum of illness, placing critical importance on the building of respectful and trusting relationships. In newborns, it is holistic and extensive care for an infant who is not going to recover. The care should include pain and symptomatic management, psychosocial support for family members, spiritual support for family members, and attention to the newborn's quality of life and best interests as determined through a culturally sensitive, negotiated, family-centered approach.^7^–^9^

Intravenous access should be kept in place to provide medication for symptom relief. These infants may be suffering from pain, seizure activity or respiratory distress. Doses of medication (that include narcotic and nonnarcotic analgesics, sedatives, hypnotics, and anticonvulsives) should be adequate to relieve these symptoms. The doses can be obtained from neonatal medications books with the help of the local pharmacist.^10^

## Who is eligible for comfort care?

The categories of newborns listed below are provided for educational purposes and can be taken as examples by the local institutions to add more eligible infants with different clinical pathologies.^11^

Newborns at the limit of viability,^12^ with extremely low birth weights and gestational ages, if no growth restriction exists. Recently, the General Presidency of Scholarly Research and Ifta, Saudi Arabia issued a legal opinion (fatwa # 231) on March 6 2008, regarding premature infants born less than six lunar months which is equal to 177 days, i.e., 25 week's gestation and two days. The legal opinion clearly stated that “*in case of infants born less than six lunar months, two specialized physicians could study the infant clinical condition and based on their opinion the infant can be offered full resuscitation if it is beneficial to the infant or to leave him/her without intervention to die but should not be deprived from nutrition or fluid*”.

Newborns with multiple congenital anomalies incompatible with prolonged life, such as trisomy 13, 15, or 18, triploidy, thanatophoric dwarfism, Potter syndrome/renal agenesis and severe lung hypoplasia, anencephaly, holoprosencephaly, inoperable heart anomalies, some cases of hypoplastic left heart syndrome, severe congenital diaphragmatic hernia with hypoplastic lungs; inoperable conjoined twins.

Newborns not responding who are deteriorating despite providing all appropriate efforts such as non-responsiveness to aggressive resuscitation, severe cases of brain injury, such as hemorrhages or leukomalacia, severe asphyxia, and multiple end-organ failure.

## Case studies

The following cases may help to demonstrate who is eligible for palliative care:

### Case 1

Baby A was a male born at 24 weeks' gestation with a birth weight of 600 g. Apgar scores were low due to chorioamnionitis and placental abruption. He developed severe respiratory distress and metabolic acidosis that required mechanical ventilation, surfactant therapy, and volume expanders. Soon after NICU admission, he developed seizure activity that required an anticonvulsant, which became almost continuous and refractory to three anticonvulsants. Brain imaging showed bilateral grade IV intraventricular hemorrhage with evidence of periventricular leukomalacia. The baby continued to have seizures most of the time for more than two weeks. Further, he developed episodes of severe bradycardia and irregular breathing while he was on low ventilatory support. Several meetings were held with his parents by the NICU staff and the pediatric neurologist. A decision was made to make him a No Cardiopulmonary Resuscitation (CPR) with comfort measures. His ventilatory settings weaned gradually while sedation increased and he died shortly thereafter.

### Case 2

Baby B was a full-term, male, born with hypoplastic left heart syndrome that was diagnosed in utero. During pregnancy, the parents were prepared and educated about the disease and its outcome. At birth, the pediatric cardiologists found the cardiac anomalies were inoperable and cardiac transplantation was not even considered because of lack of any experience for this age group at our hospital. Several meetings were held with the family and a decision was made to make him a No CPR and he was discharged home on diuretics. There was a follow-up with the family who reported his death peacefully at two months of age.

## Cultural sensitivity and spiritual support

Caring for patients across cultures requires additional skills in the areas of cultural sensitivity, awareness, knowledge and competency. Furthermore, sensitivity is a key to successful interactions between providers, patients, and families. Listening and attentiveness, combined with background knowledge, gives dignity and respect to others, a natural expression of competence.

In Saudi Arabia, Islam is the main religion; however, many caregivers are non-Muslims and may be non-Arabs. For these non-Muslims, they need to understand that Islam holds life as sacred and belonging to God and Muslims believe, as many other religions, death is only a transition between two different lives. Sacred texts and traditions, particularly the Qur'an and the Sunna “the example of the Prophet Muhammad” are the primary sources for a shared spiritual or religious response to illness among Muslims. The Qur'an repeatedly states that life is an examination. It makes no pretence that the trials and tribulations that life has to offer are significant and, at times, extremely difficult to bear; we are also reminded that these tribulations are in all cases surmountable.^13^ “*Of a surety we will test you with something of fear and hunger, loss of life…but give glad tidings to those who patiently persevere*” Qur'an 2:155-57.

Muslim parents will not accept scientific explanations on the probability of living as the only factor involved in a medical decision. Parents need to be reminded of Allah's Will, and physicians are only helping, but the ultimate outcome is in the hands of Allah. Lack of trust in physicians or hospitals and the fear of committing sins may be the reasons for parents not to accept the medical decisions, but are willing to transfer authority for such decisions to professionals or religious scholars who they trust and believe that they do the best for their baby. Furthermore, they need to be reminded that Islam assures that the destiny of their children is Paradise. “*And they who believe and whose seed follow them in faith, We cause their seed to join them (there), and We deprive them of nought of their (life's) work. Every man is a pledge for that which he hath earned*” Qur'an 52:524. This destiny is given to them as a way of honoring them for the patience they show upon losing these little ones. It is also a sign of the magnificent and the all-embracing mercy of Allah that covers everything and everyone. They should be reminded to pray more frequently that Allah alleviates the sufferings of their dying children because Allah increases the rewards of parents who show patience in face of tribulations.

When a Muslim dies, ideally, the face of the person who has died should be turned towards Makkah, but in hospital turning the face towards the right should be sufficient. The arms and legs should be straightened and the mouth and eyes closed and the body covered with a sheet. A baby dying at or even before birth has to have a name. The corpse is ritually bathed before burial. A stillborn baby can be dealt with in the same manner as a live birth. Following the child's death, there are religious, social and bereavement rituals that are explained in detail by al-Shahri and al-Khenaizan.^14^,^15^ The bereavement care should include not only the parents but also siblings, grandparents, other relatives, friends, nurses, physicians, and other staff members who have cared for the child.

## Strategy for developing a palliative care program

Development should focus on the concept that palliative care is a part of the continuum of care, including parent choice and integrity throughout. The lack of continuity of care during the stages of death and dying with newborns is the first issue to be raised by professionals in order to discuss the need for a multidisciplinary palliative care team to effect continuity of care through to end-of-life.^16^ Furthermore, a comprehensive approach to palliative care for seriously ill neonates, which would begin well before the neonate is imminently dying, should be integrated with other treatment modalities earlier in the disease course. One of the major defects in care is inadequate communication by the staff with parents and other members of the family. Thus, education of healthcare professionals in palliative care and instituting the model will accomplish the goals of the program. This can be achieved by developing educational tools and guidelines for the palliative care program, which could be obtained from the experiences of others in this field.^17^ There are seven stages in the launch and a quality run in the development of a neonatal palliative care program. These are: building the case for a hospital-based palliative care program, designing an operational plan, presenting the business plan, implementing palliative care services, measuring program quality and impact, marketing the palliative care program and sustaining and growing the program.

The program should include well-experienced nurses who play clinical leadership roles on the palliative care team, as well as neonatologists, program administrators, educators, and researchers.^18^ Once the program is implemented, it should be evaluated in 6-month increments and the input of parents should be taken into consideration. Outcome measures can be used to assess deficiences and determine necessary changes to be corrected by the team.

## Barriers to neonatal palliative care

Despite the existence of a universal protocol in palliative care for dying babies and their families, provision of this type of care remains ad hoc in contemporary neonatal settings. Several barriers have been reported and they vary from one place to another. The input of nurses looking after dying neonates was the main source for identifying most of these barriers. Several themes emerged from the literature indicating that barriers for neonatal nurses may be attitudinal, clinical, educational, institutional, regulatory, and financial. Australian NICU nurses identified the following barriers: (1) inadequate staffing to support palliative care practice; (2) a physical environment that is not conducive to palliative care practice; and (3) technological imperatives and parental demands.^19^

The Neonatal Palliative Care Attitude Scale (NPCAS) surveys the following key barriers: (a) neonatal death viewed as a failure; (b) adjustment from a curative to palliative care approach; (c) difficult communication with parents of dying neonates; (d) previous and traumatic exposure to neonatal death; (e) conflicts among providers around end-of-life decision making; (f) NICU environment; (g) lack of support for nurses providing end-of-life care; and (h) lack of formal training for nurses.^11^ Solving these barriers in a timely manner will maintain the continuation of the program.

## Conclusion

Establishing neonatal palliative care programs at our hospitals is one of the vital needs in our institutions. These programs will provide crucial support for parents in going smoothly through their stressful events with a feeling that they are not abandoned. Speaking to parents about palliative care is not an easy task. Parents need to hear that physicians are willing to continue to provide the best medical care possible for their infants. They need to know that this continuing care will include frequent assessments by care givers and social workers in addition to adjusting medications so that their infant is comfortable. It is important to offer the ability to have a second opinion and an ethics or religious consultation. The language used should be lay person language to clarify medical terms, and the terms “withdrawal of treatment” or “withdrawal of care” should be avoided. It is also important not to give them the feeling that they are responsible for the infant's death, but they should understand that they cannot change the situation and the only thing can be offered is to support the infant's short-life with comfort and dignity.
